# Supplementary material for: Color Rendering Index over 95 Achieved by Using Light Recycling Process Based on Hybrid Remote-Type Red Quantum-Dot Components Applied to Conventional LED Lighting Devices
Source: Nanomaterials (Basel). 2023 Sep 15;13(18):2560. doi: 10.3390/nano13182560 (PMC10534905; doi:10.3390/nano13182560)

Supplementary Materials

Color Rendering Index over 95 Achieved by Using Light Recycling Process Based on Hybrid Remote-type Red Quantum-dot Components Applied to Conventional LED Lighting Devices

Eunki Baek*, Boseong Kim*, Sohee Kim*, Juyeon Song*, Jaehyeong Yoo*, Sung Min Park, Jong-Min Lee, Jae-Hyeon Ko**

School of Semiconductor·Display Technology, Nano Convergence Technology Center, Hallym University, Chuncheon 24252, Gangwondo, Korea

***** These authors contributed equally to this work.

****** Correspondence: hwangko@hallym.ac.kr

Table S1. 12 different configurations depending on the type of the reflector, the QD film, and the number of QD caps.

QD film type / reflector	Ring-type	Wall-type	
			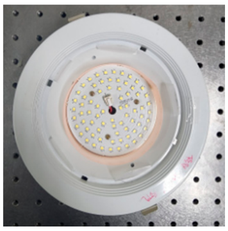 <wall-type>
Mirror Reflector	Hybrid 1	Hybrid 2	
Diffusion Reflector	Hybrid 3	Hybrid4	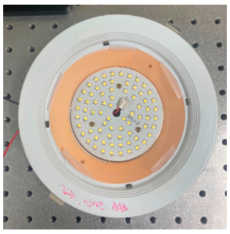 <ring-type>

(a) No QD caps: Only QD films

QD film type / reflector	Ring-type	Wall-type	
			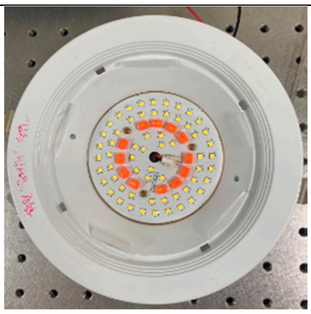
Mirror Reflector	Hybrid 5	Hybrid 6	
Diffusion Reflector	Hybrid 7	Hybrid 8	

(b) 12 QD caps adopted

QD film type / reflector	Ring-type	Wall-type	
			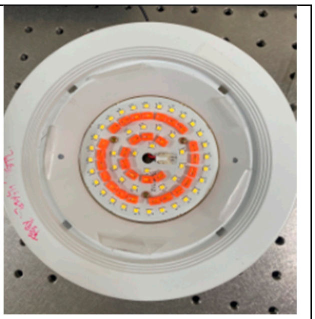
Mirror Reflector	Hybrid 9	Hybrid 10	
Diffusion Reflector	Hybrid 11	Hybrid 12	

(c) 29 QD caps adopted

Figure S1. Photos of experimental components used in the present study.

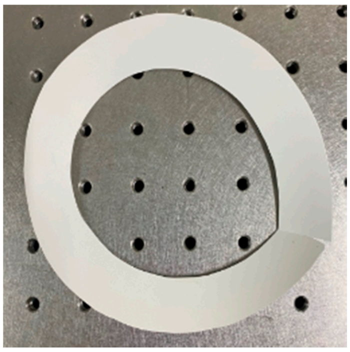

(a) Diffusion Reflector

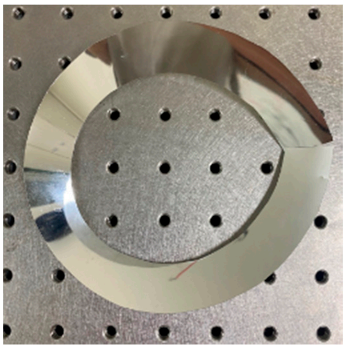

(b) Mirror Reflector

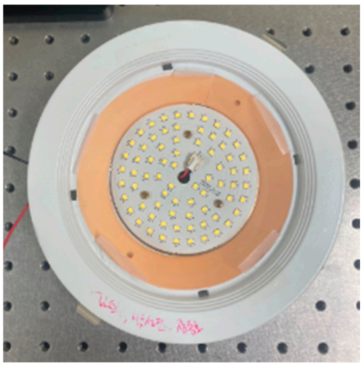

(c) Ring-type QD film

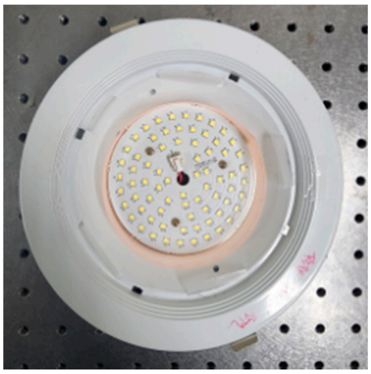

(d) Wall-type QD film

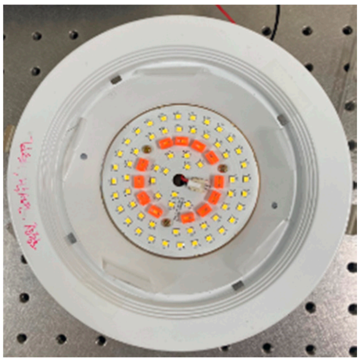

(e) 12 QD caps on the 3rd line

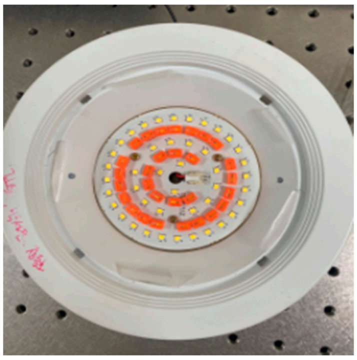

(f) 29 QD caps on the 2nd and 4th lines

Figure S2. The dependence of (a) the CCT and (b) the Ra CRI on the type of reflector and the QD concentration in the QD wall film.

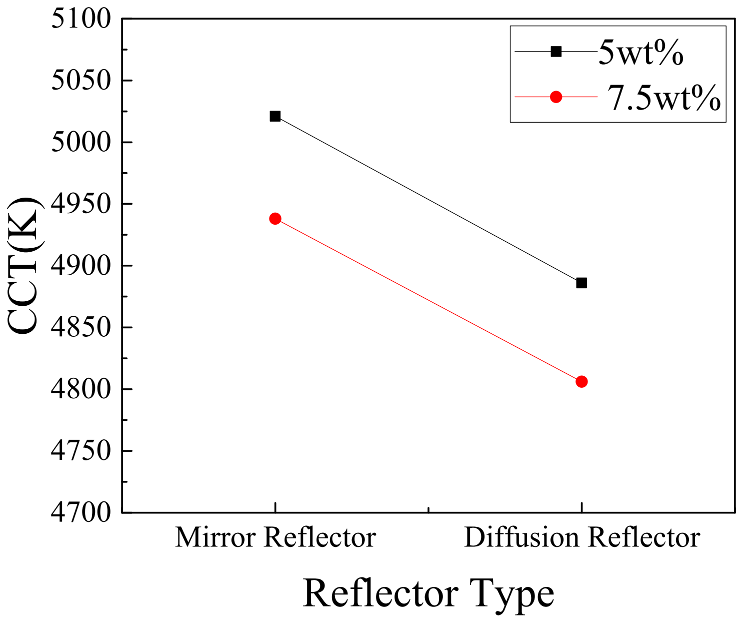

(a)

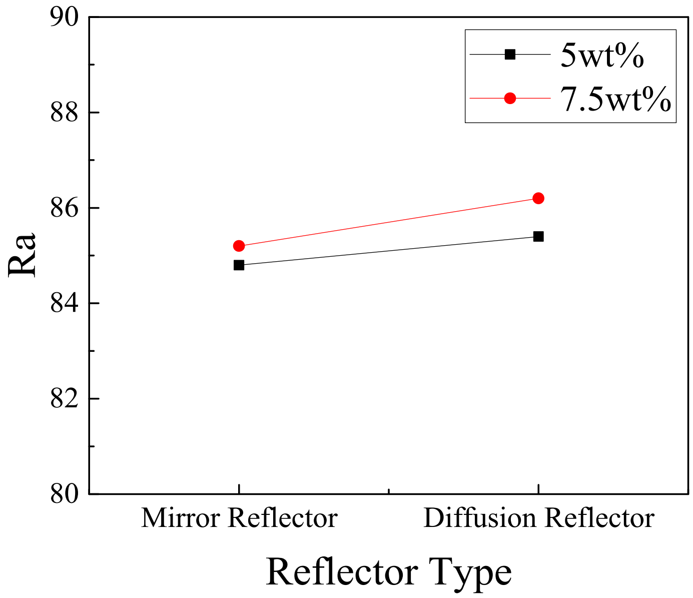

(b)

Figure S3. Photometric and color properties of the "Hybrid 1~4(Only QD films)" configurations: (a) the luminance, (b) the CCT, (c) the Ra, and (d) the R9 CRI values.

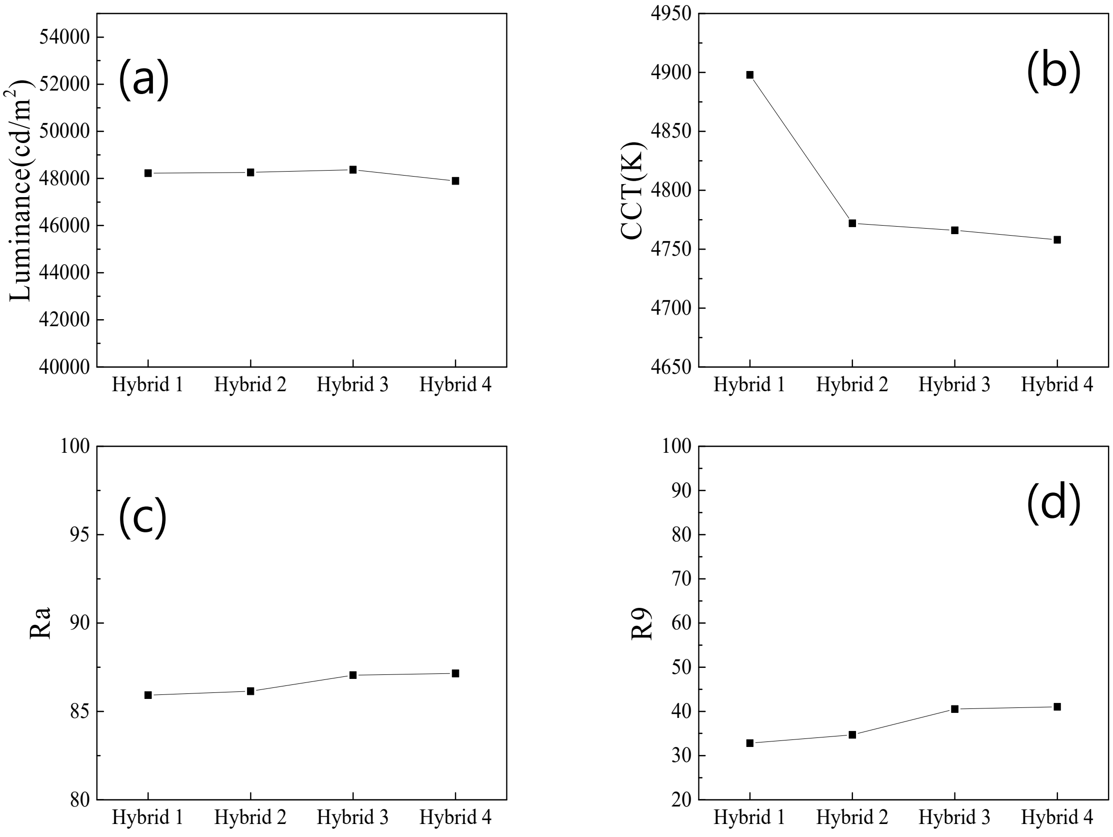

Figure S4. The individual CRI values of (a) the white LED illumination without any QD components and (b) that with 29 QD caps together with the ring configuration (Hybrid 11 configuration).

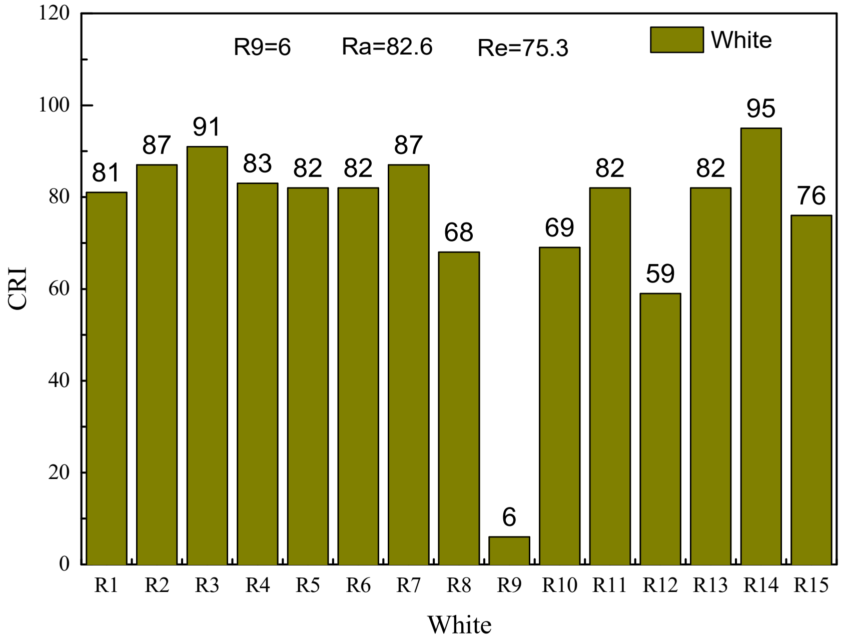

(a)

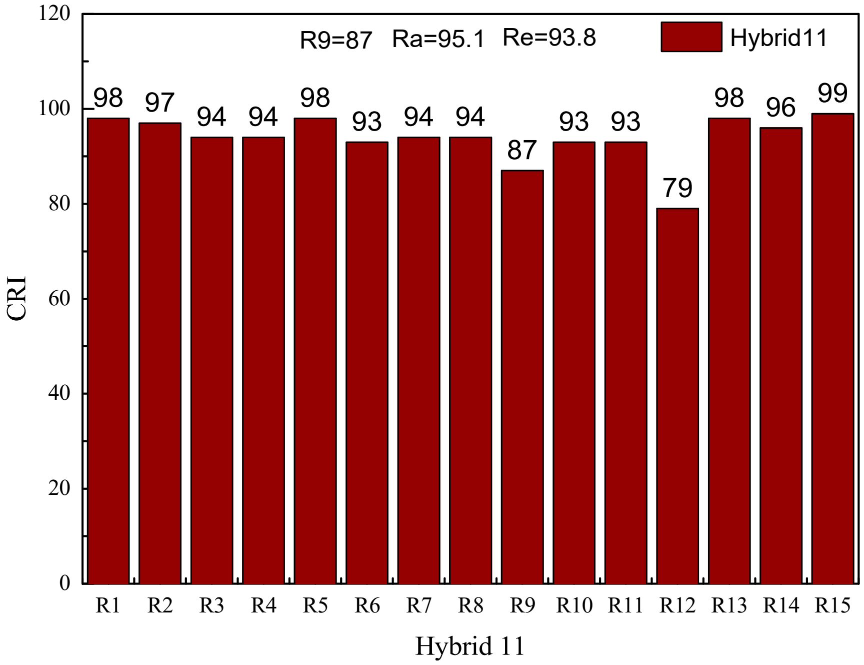

(b)

Figure S5. (a) The luminance, (b) the CCT, (c) the Ra, and (d) the R9 of "Hybrid 1~4" configurations depending on the combination of optical films placed on the PC diffuser.

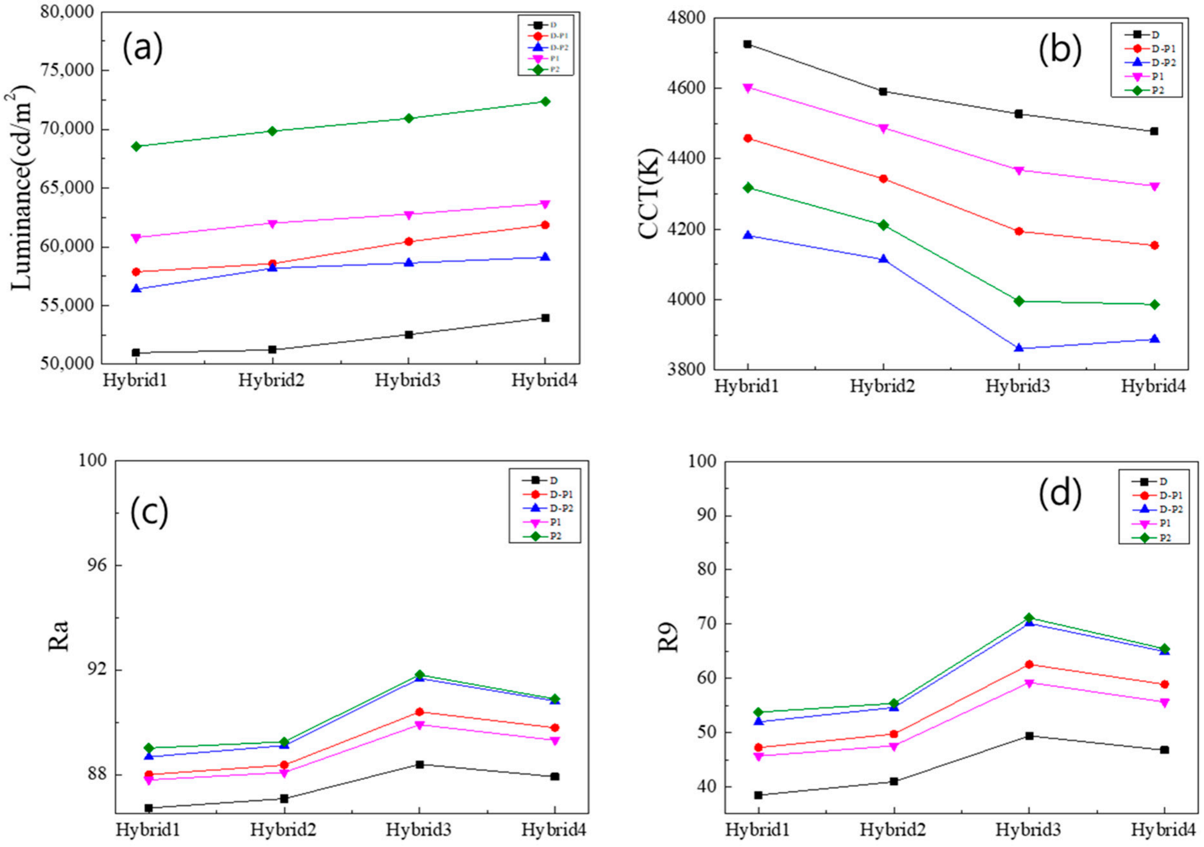

Figure S6. (a) The luminance, (b) the CCT, (c) the Ra, and (d) the R9 of "Hybrid 5~8" configurations depending on the combination of optical films placed on the PC diffuser.

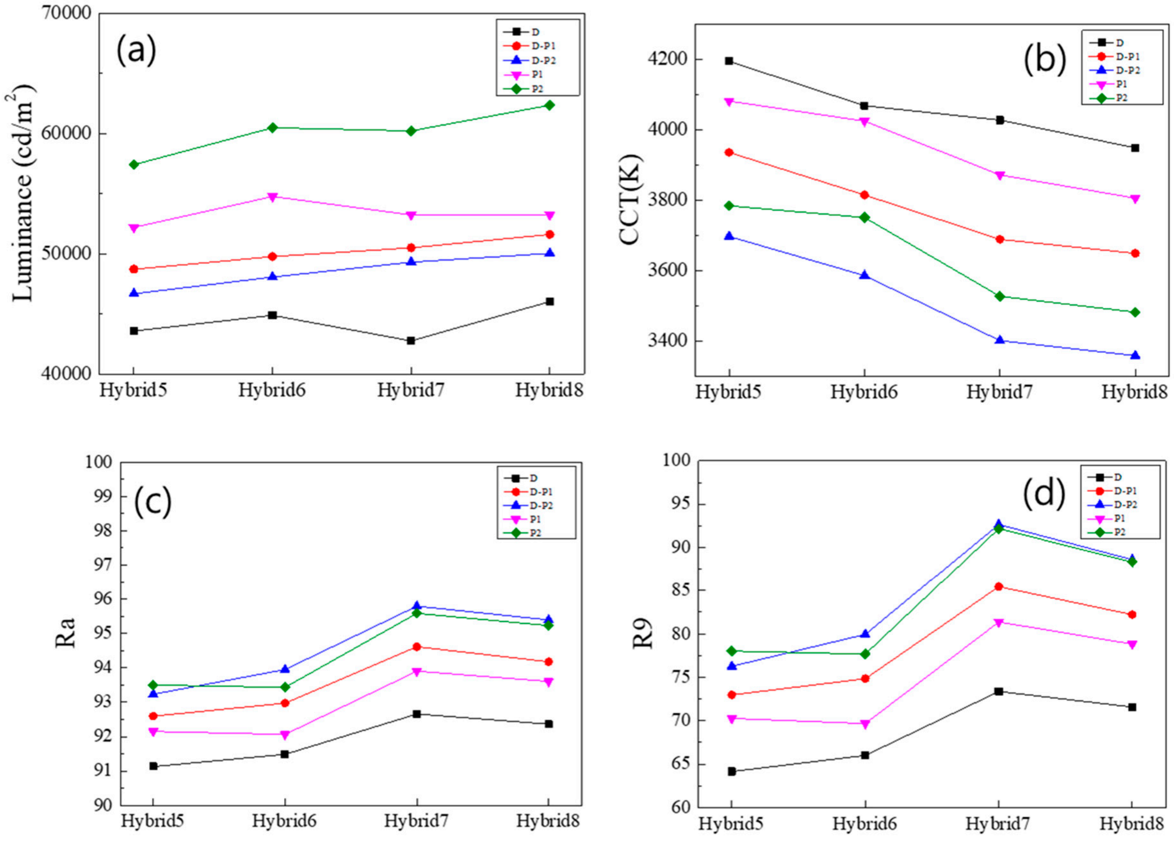

Figure S7. (a) The luminance, (b) the CCT, (c) the Ra, and (d) the color coordinates on the chromaticity diagram of "Hybrid 11" configuration depending on the combination of optical films placed on the PC diffuser.

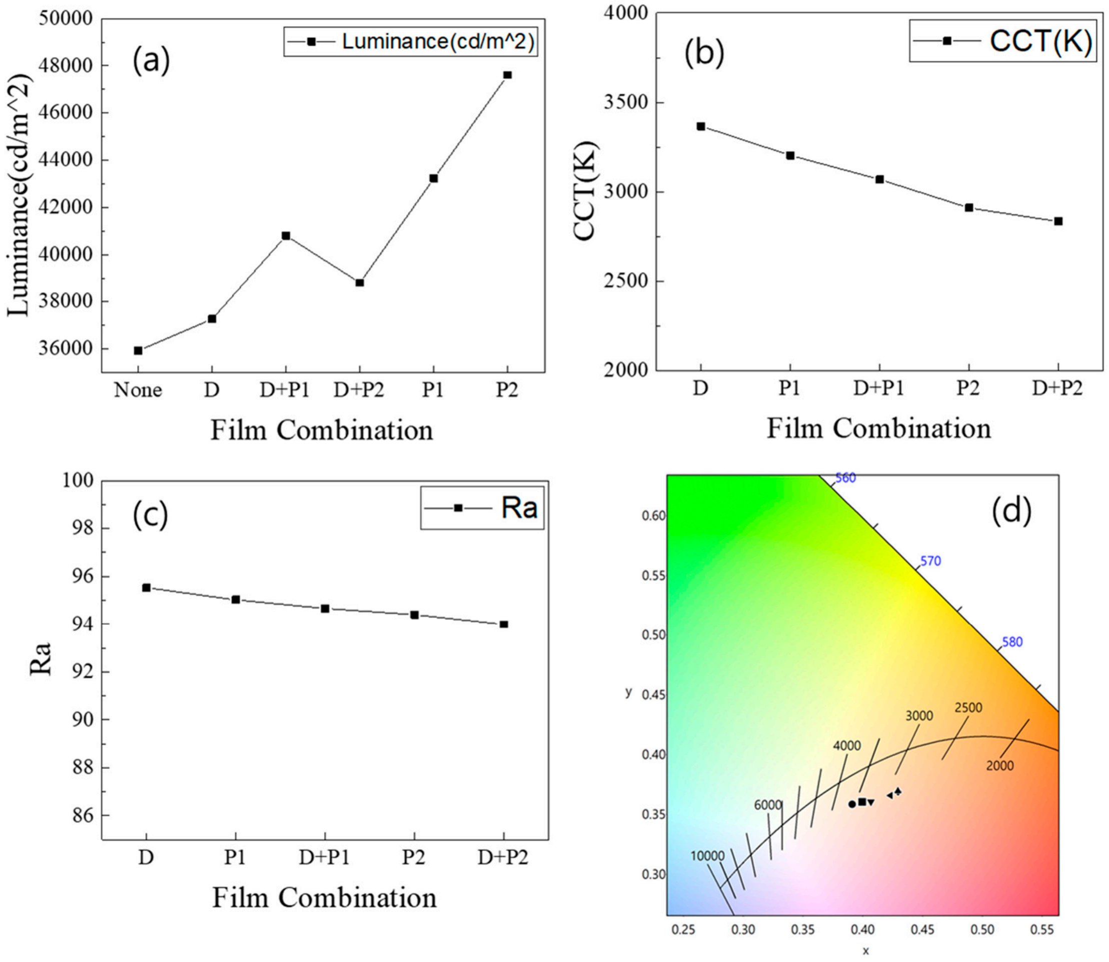

Supplement: Supplementary file 1 [file nanomaterials-13-02560-s001.zip › nanomaterials-2594956-supplementary.pdf]
